# Effects of Fructose on Features of Steatotic Liver Disease in HepG2 Cells

**DOI:** 10.3390/nu17172762

**Published:** 2025-08-26

**Authors:** Matthew Thomas Howes, Jessie King, Rhonda Joy Rosengren

**Affiliations:** Department of Pharmacology and Toxicology, University of Otago, Dunedin 9016, New Zealand; jessie.king@otago.ac.nz (J.K.); rhonda.rosengren@otago.ac.nz (R.J.R.)

**Keywords:** metabolic disfunction-associated steatotic liver disease (MASLD), HepG2 cells, fructose, de novo lipogenesis

## Abstract

Background/Objectives: Metabolic (dysfunction)-associated steatotic liver disease (MASLD), the hepatic consequence of metabolic syndrome, affects 30% of the global population. Studies in animals and humans investigating the effect of fructose on MASLD present conflicting findings, while in vitro methods often fail to add meaningful evidence due to acute exposures (<72 h) and non-physiological concentrations. This study aimed to determine the effect of fructose on triglyceride (TG) accumulation in HepG2 cells following acute and chronic exposures and assess its effect on the expression of genes related to de novo lipogenesis (DNL). Methods: TG concentration was measured after 48 h in response to fructose (20 mM) or glucose (20 mM), with or without a fatty acid mixture (oleic acid/palmitic acid 110 µM/55 µM), in low (5.5 mM)- and high (25.5 mM)-glucose media. To model chronic exposure, cells were maintained in fructose, glucose, or fatty acids for 28 days and the TG concentration was determined every 7 days. The effect of fructose on DNL regulators (*SREBPF1*, *NR1H3*, *FASN*, and *ACACA*) was determined using qPCR. Results: Neither fructose nor glucose, with or without fatty acids, changed the TG levels in cells at 48 h and the media glucose concentration had no effect on this result. Similarly, fructose did not increase TG levels after 28 days. While fructose and glucose did not affect key DNL genes at 6 h, the fatty acid mixture reduced *FASN* by 41%. Conclusions: This study shows that fructose did not significantly impact TG synthesis or DNL gene expression in the HepG2 cell model. Future studies should consider using primary human hepatocytes or more complex in vitro models.

## 1. Introduction

Metabolic (dysfunction)-associated steatotic liver disease (MASLD), formerly known as non-alcoholic fatty liver disease, is prevalent in 30% of the global population [1,2]. MASLD is diagnosed when there is evidence of hepatic steatosis (lipid accumulation) alongside at least one cardiometabolic risk factor. Risk factors for MASLD include genetic predisposition, metabolic health, and dietary choices [2]. In liver cells, the accumulation of highly saturated triglycerides rich in palmitic acid and stearic acid is a hallmark of MASLD and contributes to lipotoxicity, leading to fibrogenesis, inflammation, and cell death [3]. Fructose, specifically, has been investigated extensively as a dietary contributor since it was first noted by Plini the Elder that feeding geese with dried figs increased the size of their liver [4]. This association resurfaced when high-fructose, but not high-glucose, sweetened beverages were reported to increase hepatic lipogenesis in adults [5]. Meta-analyses provided a less clear picture where fructose only increased intra-hepatic lipid content when consumed in excess of 100 g/day [6,7], while evidence in rodent models implicated fructose as a potent lipogenic substrate [8,9]. However, principle component analyses of transcriptomes comparing human and rodent livers showed that they clustered by species (rodent or human) rather than liver state (steatotic or healthy) [10]. Conclusions from rodent models that poorly resemble human disease progression should therefore be interpreted with caution. Mechanistically, fructose is proposed to upregulate triglyceride (TG) levels in hepatocytes by either the products of fructose metabolism acting as direct substrates for de novo lipogenesis (DNL), or by fructose itself upregulating key transcription factors and enzymes involved in DNL (for a review, see Herman and Samuel 2016) [11].

The HepG2, human hepatocellular carcinoma, cell line is used in 76% of in vitro studies using hepatic cells and has a relatively high sequence homology with hepatocytes from liver tissue (80–82%) [12,13,14]. In this cell line, the association between fructose and steatosis is not consistent: some studies report concentration- and time-dependent increases in TG accumulation when exposed to fructose [15,16,17], while other groups report no changes in TG levels [18,19,20]. Methodological differences may account for such discrepancies, yet key factors such as the glucose concentration in the culture media are often not controlled. Additionally, the maximum exposure period of 72 h does not model how fructose is consumed as part of a typical diet, that is, multiple times a day for years.

This study therefore aimed to perform tightly controlled experiments to determine the effect of fructose on TG accumulation in HepG2 cells under both acute (48 h) and chronic (28 d) exposure. Furthermore, the experiments were performed in high- and low-glucose media with and without fatty acids. Additionally, the effect of fructose on selected transcription factors and enzymes related to DNL was determined.

## 2. Materials and Methods

### 2.1. Chemicals

Sodium oleate, palmitic acid, D-(−)-fructose, D-(+)-glucose, trichloroacetic acid, sulforhodamine B, NP-40, the Pierce^TM^ BCA protein assay kit, and Dulbecco’s modified Eagle medium—low glucose—were purchased from Sigma Aldrich (Auckland, New Zealand). Bovine serum albumin fraction V, penicillin–streptomycin, TrypLE™ express enzyme, the Infinity™ triglyceride liquid stable reagent, and Nile red were purchased from Thermo Fisher Scientific (Auckland, New Zealand). The TG standard was purchased from Roche Diagnostics (Auckland, New Zealand). Foetal bovine serum was purchased from medi’Ray (Auckland, New Zealand). Buffer RLT was purchased from Qiagen (Hilden, Germany). The GENEzol^TM^ TriRNA Pure kit, Geneaid DNase I set, Quantabio qScript^TM^ XLT cDNA Supermix, and Quantabio PerfeCTa SYBR Green FastMix were purchased from dnature (Gisborne, New Zealand). Primers were designed using the National Center for Biotechnology Information Primer-Blast tool and purchased from Integrated DNA Technologies (Coralville, IA, USA).

### 2.2. Cell Culture

The HepG2 cell line was purchased from the American Type Culture Collection (Manassas, VA, USA) and maintained at 37 °C with 5% cardon dioxide and 95% humidity. Cells were cultured in Dulbecco’s modified Eagle medium (DMEM)—low glucose (5.5 mM glucose)—supplemented with 5% (*v*/*v*) foetal bovine serum and a penicillin (100 U/mL) and streptomycin (0.1 mg/mL) mixture. Cells were sub-passaged once every 2–3 days and experiments were conducted on cells between passages 5 and 20.

### 2.3. Ensuring Fatty Acid Concentrations Were Not Toxic

High concentrations of free fatty acids including oleic acid and palmitic acid, a combination regularly used as a positive control in TG accumulation assays, can be cytotoxic in cultured hepatocytes, even at 660/330 µM [21,22,23]. To ensure that the fatty acid concentrations used as a positive control in experiments were non-toxic, cell viability was determined after 72 h. Specifically, HepG2 cells were exposed to varying concentrations of bovine serum albumin (BSA)-conjugated oleic acid, palmitic acid, or a combination of both. BSA-conjugated standards of oleic acid and palmitic acid were prepared in 0.1 N NaOH and heated at 70 °C for 1 h. Once dissolved, the standards were diluted 1:2 in 10% BSA solution and gently rocked at 37 °C for 10 min to conjugate. Final concentrations were 120 mM oleic acid and 40 mM palmitic acid in 5% BSA, and aliquots were stored at −20 °C until use. HepG2 cells grown in low-glucose DMEM were plated at 3.5 × 10^5^ cells per well in 6-well plates and incubated for 24 h to adhere. Cells were then exposed to vehicle (0.01% BSA/0.11 mM NaOH), or a 2:1 combination of oleic acid/palmitic acid (1.5 μM–3 mM) for 72 h. The SRB assay was then performed to determine cell viability [24]. The combination of 110 µM oleic acid and 55 µM oleic acid (i.e., 165 µM combined fatty acids) was the highest concentration that did not reduce cell number at 72 h (Supplementary Figure S1) and was thus used as a positive control for all subsequent experiments.

### 2.4. TG Assay

HepG2 cells were plated in 6-well plates at 3.5 × 10^5^ cells/well in low-glucose DMEM and left for 24 h to adhere. Wells were then treated with the indicated concentrations of fructose and incubated for 72 h. Cell harvesting and sample processing to determine the TG concentration was modified from a previous method [16]. Following incubation, the culture media were aspirated, and cells were washed with PBS. Cells were detached using a scraper and suspended on ice in 5% NP-40. Samples were then briefly sonicated (30% ampule; 21 s on, 10 s off) before centrifugation at 20,817× *g* for 8 min. The protein content of each sample was then determined using a Pierce^TM^ BCA protein assay kit with a BSA standard curve. Samples were then heated twice to 90 °C for 3 min and stored at −20 °C for at least 24 h. To determine the TG concentration, samples were thawed to room temperature and mixed by manually pipetting samples up and down and then vortexed. Next, 10 µL of each sample was added to a 96-well plate and incubated with 150 µL of the Infinity triglyceride reagent for 10 min at 37 °C. The absorbance at 510 nm was read using a Bio-Rad Benchmark Plus™ microplate spectrophotometer (Bio-Strategy, Auckland, New Zealand) and used to determine the TG concentration relative to a TG standard curve. The TG standard curve was prepared at 0, 3.125, 6.25, 12.5, 25, 50, 100, and 172 mg/dL. A representative correlation coefficient was 0.9979 with a regression equation of TG (mg/dL) = (absorbance at 510 nm − 0.02255)/0.004875. Concentrations were normalised to total cell protein which was determined relative to a BSA standard curve. The BSA standard curve used BSA concentrations of 0, 50, 100, 150, 200, 250, 300, 350, 400, 450, and 500 µg/mL. A representative correlation coefficient was 0.9894 with a regression equation of BSA (µg/mL) = (absorbance at 562 nm − 0.01577)/0.0007929. The normalised data were expressed as a percentage of the control.

### 2.5. Acute Fructose Exposure in Low- and High-Glucose Media

Cell culture media can contain up to 25 mM glucose, whereas a physiological concentration is between 4 and 7 mM [25]. To investigate what effect this has on TG accumulation in response to fructose exposure, HepG2 cells cultured in low (5.5 mM)- or high (25.5 mM)-glucose DMEM were seeded in 6-well plates at 5 × 10^5^ cells/well and incubated for 24 h to allow cells to adhere. Cells were then treated with fructose (20 mM), glucose (20 mM), a combination of fatty acids (110 µM oleic acid, 55 µM palmitic acid), fructose + fatty acids, or glucose + fatty acids. After 48 h, cell lysates were harvested, and the TG concentration was determined.

### 2.6. Nile Red Assay

The fluorescent dye Nile red (9-diethylamino-5H-benzo[alpha]phenoxazine-5-one) binds to neutral lipid droplets and was used to confirm the results from the TG assay. HepG2 cells plated in low (5.5 mM)- or high (25.5 mM)-glucose DMEM at 3 × 10^4^ cells/well in black-walled 96-well plates were left to adhere for 24 h and then treated for 48 h. Media were then aspirated and cells were washed three times with PBS and incubated with Nile red (10 µM) for 15 min at 37 °C, protected from light. The plate was then washed three times with PBS. The relative fluorescent units were determined with excitation set at 488 nm and emission set at 565 nm, using a SpectraMax i3x multi-mode microplate reader (Bio-Strategy, Auckland, New Zealand).

### 2.7. Chronic Fructose Exposure

Acute exposure to fructose for up to 72 h is a common protocol to determine the effect on TG accumulation, yet fructose is consumed by most of the population multiple times a day, for years at a time. To address this, a chronic exposure model was developed. HepG2 cells were maintained and passaged in low-glucose DMEM supplemented with vehicle (0.01% BSA/0.11 mM NaOH), fructose (20 mM), glucose (20 mM), or a combination of fatty acids (110 µM oleic acid, 55 µM palmitic acid) for 28 days. At days 7, 14, 21, and 28, cells from each condition were plated at 1 × 10^6^ cells/well in 6-well plates and left to adhere for 24 h. Cell lysates were then harvested, and the TG concentration was determined. Each treatment flask was passaged every two to three days and cell confluency <80% was maintained. Low-passage cells were used over the 28-day exposure (P5-17). Cell morphology was also monitored every two to three days as an indicator of cell health, and a change in morphology was only detected in the fatty acid group due to lipid droplet accumulation.

### 2.8. qPCR

TG synthesis and breakdown is tightly regulated in cells [26], meaning transient changes in gene expression related to DNL may be missed in assays that determined TG accumulation in cells after 24 h. Therefore, qPCR was used to determine the expression level of key DNL genes following 6 h exposure. HepG2 cells in low-glucose DMEM were plated in 10 cm Petri dishes at 2 × 10^6^ cells/dish and incubated for 24 h to allow cells to adhere. Cells were then treated with vehicle (0.01% BSA/0.11 mM NaOH), fructose (20 mM), glucose (20 mM), or a combination of oleic acid and palmitic acid (110 µM, 55 µM) for 6 h. Media were removed, and cells were washed twice with cold PBS and harvested in 600 µL of Buffer RLT using a cell scraper. The lysate was then syringed 20 times with a 20-gauge needle and stored at −80 °C until used. RNA extraction was performed using a TriRNA Pure kit and the RNA concentration and quality were determined by absorption at 260/280 nm and 260/280 nm using a DeNovix DS-11 spectrophotometer (dnature, Gisborne, New Zealand). cDNA was then synthesised by incubation with DNase I followed by PCR with qScript XLT cDNA supermix, yielding 25 ng/µL cDNA. Primers were designed for the transcription factors liver x receptor-α (*NR1H3*) and sterol regulatory element-binding protein 1-c (*SREBF1*) and enzymes acetyl-CoA carboxylase-α (*ACACA*) and fatty acid synthase (*FASN*) using the National Center for Biotechnology Information primer design tool, and the efficiency of each was determined across a range of cDNA dilutions (Table 1).

A total of 4 ng of each cDNA preparation and 500 nM of primer pairs were combined with Quantbio PerfeCTa^®^ SYBR^®^ Green FastMix^®^, and qPCR was run using a Mic qPCR thermal cycler (Bio Molecular Systems, Gold Coast, Australia). The run profile was as follows: hold at 95 °C for 2 min, followed by 40 cycles of 95 °C for 10 s to denature, and 60 °C for 30 s to anneal. Cycle threshold (Ct) values were reported as a fold-change relative to the vehicle control and expressed relative to the housekeeper gene β-actin (*ACTB*) using the ΔΔCt method [27].

### 2.9. Statistical Analysis

Data are expressed as the mean ± SD, and statistical testing was conducted using GraphPad Prism (version 10.2.3) (Boston, MA, USA) with three independent biological replicates performed in duplicate unless specified. Data from the chronic exposure experiments were analysed using a two-way repeated-measures ANOVA, and differences between groups were determined using a Tukey multiple-comparison post hoc test. Data from all other experiments were analysed using a one-way ANOVA, and the differences between groups were determined using a Bonferroni multiple-comparison post hoc test. *p* < 0.05 was the minimum requirement for a statistically significant difference.

## 3. Results

### 3.1. Effect of Fructose on TG and Lipid Accumulation

**Figure 1:**
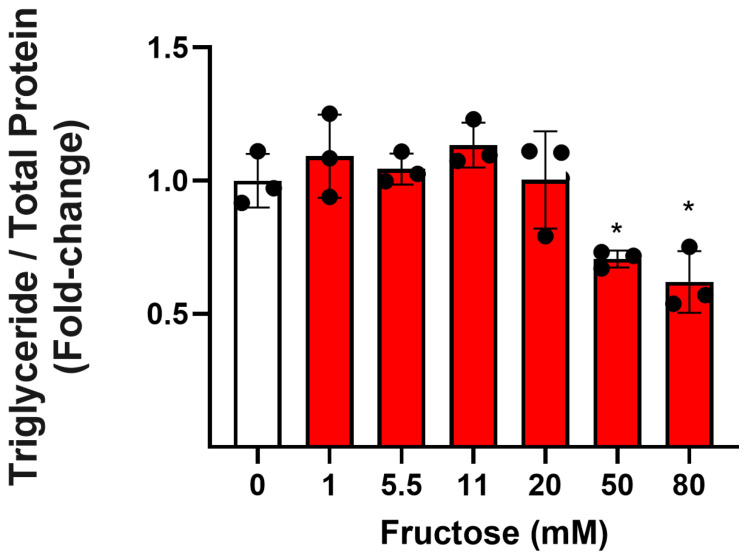
Effect of fructose on triglyceride (TG) concentrations. HepG2 cells plated in 6-well plates at 3.5 × 10^6^ cells/well were exposed to a range of fructose concentrations for 72 h. Cell lysates were incubated with the Infinity triglyceride reagent, and absorbance at 510 nm was used to determine the TG concentration relative to a standard curve using linear regression. TG concentrations were normalised to total cell protein and expressed as a fold-change compared to the control. Columns represent the mean ± SD from three independent experiments performed in duplicate and expressed as fold-change. Statistical significance was analysed using a one-way ANOVA with differences between groups determined using a Bonferroni multiple-comparison post hoc test. * significantly decreased compared to the control, *p* < 0.05.

To determine the effect of varying concentrations of fructose on TG concentrations, HepG2 cells were exposed to a range of fructose concentrations for 72 h. The TG concentration as a percentage of total protein in cell lysates was significantly reduced by 28.2% at 50 mM and 38% at 80 mM compared to the vehicle control, *p* < 0.05 (Figure 1). Importantly, total cell protein concentration was not changed by any concentration of fructose (Supplementary Figure S2).

**Figure 2:**
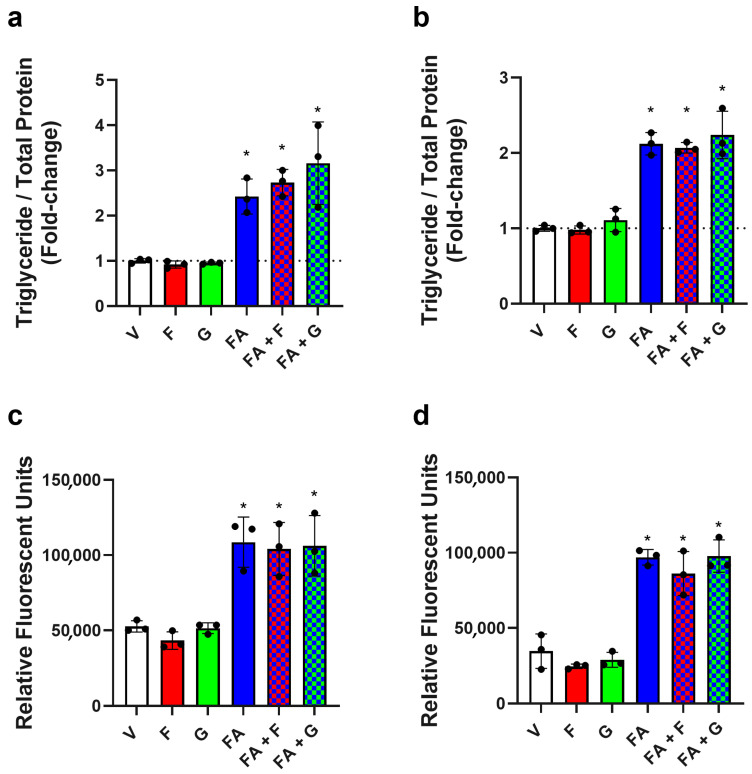
Effect of sugars alone, or in combination with a fatty acid mixture, on TG concentration and lipid accumulation. HepG2 cells cultured in (**a**,**c**) low-glucose media (5.5 mM) or (**b**,**d**) high-glucose media (25.5 mM) were treated with vehicle (V), fructose (F, 20 mM), or glucose (G, 20 mM) alone or in combination with oleic and palmitic acid (FA, 110 µM/55 µM) for 48 h. The TG concentrations in cell lysates were determined using the Infinity triglyceride reagent normalised to total cell protein (**a**,**b**). The horizontal dashed line (**a**,**b**) indicates the level of the vehicle-treated control. Lipid accumulation was determined by incubation with Nile red dye (10 µM), and fluorescence intensities were read using a SpectraMax i3x multi-mode microplate reader with an excitation wavelength of 488 nm and emission wavelength of 565 nm (**c**,**d**). Columns represent the mean ± SD from three independent replicates performed in duplicate. Statistical significance was analysed using a one-way ANOVA with differences between groups determined using a Bonferroni multiple-comparison post hoc test. * significantly increased compared to V, F, and G, *p* < 0.05.

To determine the effect of background media glucose concentration on TG accumulation, cells were cultured in low (5.5 mM)- or high (25.5 mM)-glucose DMEM and exposed to fructose (20 mM) or glucose (20 mM). No changes in TG content were observed, regardless of the background media glucose concentration or experimental fructose or glucose exposure (Figure 2a,b). Additionally, to determine if fructose could indirectly upregulate lipogenesis, cells were exposed to fructose in the presence of a fatty acid mixture. The fatty acid combination alone increased the TG concentration in cell lysates by 2.4-fold in cells cultured in low-glucose DMEM and 2.1-fold in those cultured in high-glucose DMEM, and the addition of fructose or glucose did not change this effect (Figure 2a,b). Furthermore, there was no change in cell viability compared to the vehicle following any treatment combination (Supplementary Figure S3a,b).

The Infinity triglyceride reagent assay uses a series of enzyme-coupled reactions to detect TGs in intracellular lipid droplets. To validate the acute exposure results, the fluorescence intensity of Nile red, a dye that accumulates in neutral lipid droplets, was determined. Similar trends were seen as in the Infinity TG assay where, for cells cultured in low (Figure 2c)- and high (Figure 2d)-glucose media, neither fructose nor glucose affected Nile red intensity compared to control. However, the experimental protocol could detect changes, as the fatty acid combination increased the Nile red intensity in the low-glucose group by 206% (Figure 2c) and in the high-glucose group by 280% (Figure 2d). Fructose and glucose in combination with the fatty acids had no impact on Nile red intensity in either high- or low-glucose media.

### 3.2. Chronic Model

**Figure 3:**
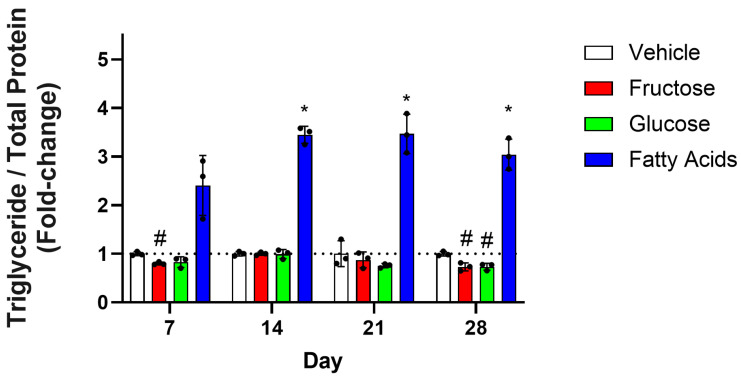
Effect of sugars on TG concentrations over 28 days. HepG2 cells were cultured in vehicle (0.01% BSA/0.11 mM NaOH), fructose (20 mM), glucose (20 mM), or a combination of fatty acids (110 µM oleic acid, 55 µM palmitic acid) for 28 days. Every seven days, the TG concentration was determined in cell lysates using the Infinity triglyceride reagent and normalised to total protein as determined using a Piece BCA reagent kit. Columns represent the mean ± SD from three independent replicates and are expressed as a fold-change compared to vehicle. The horizontal dashed line indicates the level of the vehicle-treated control. Statistical significance was analysed using a two-way repeated-measures ANOVA and differences between groups were determined using a Tukey multiple-comparison post hoc test. * significantly increased compared to the vehicle control; # significantly decreased compared to the vehicle control, *p* < 0.05.

To more closely model a Western diet, the TG concentration was determined in HepG2 cells that were grown and passaged in fructose, glucose, or a fatty acid combination for 7, 14, 21, and 28 days. At day 7, fructose reduced the concentration of TGs by 20%, and after 28 days, both fructose and glucose reduced the concentration of TGs by 27% (Figure 3). The fatty acid mixture positive control confirmed that this protocol could elicit significant TG accumulation after 14 days.

### 3.3. Effect of Fructose on Genes Involved in DNL

**Figure 4:**
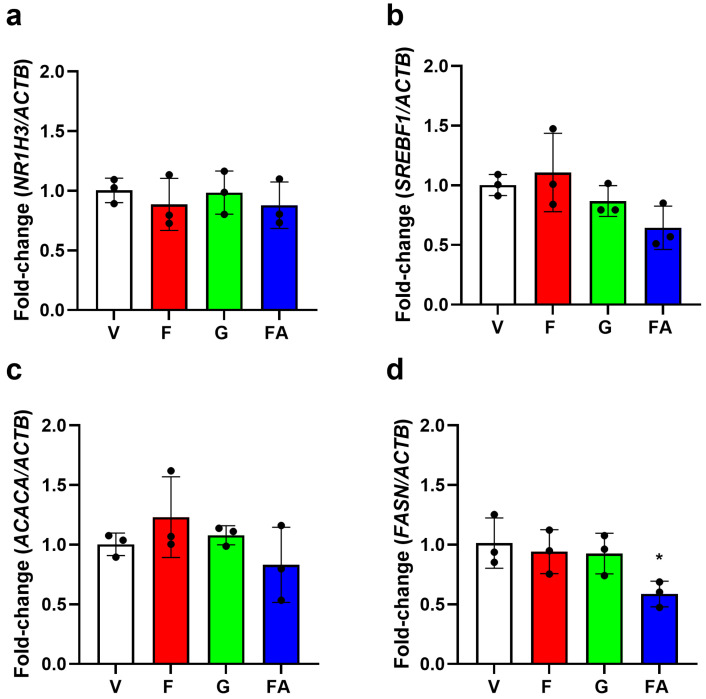
Effect of sugars on gene expression of de novo lipogenesis transcription factors and enzymes. HepG2 cells were exposed to vehicle (V), fructose (F, 20 mM), glucose (G, 20 mM) or fatty acids (FA, 110 µM oleic acid, 55 µM palmitic acid) for 6 h. RNA was harvested from cell lysates, and cDNA synthesised. The expression level of *NR1H3* (**a**), *SREBF1* (**b**), *ACACA* (**c**) and *FASN* (**d**) was determined using qPCR. Data from three independent replicates performed in duplicate were normalised to the housekeeper gene *ACTB* and columns represent a mean ± SD relative to the vehicle control. Statistical significance was analysed using a one-way ANOVA with differences between groups determined using a Bonferroni multiple-comparison post hoc test. * significantly decreased compared to control, *p* < 0.05.

To determine if sugars induced transient changes in the expression levels of genes related to DNL, the expression levels of the transcription factors *NR1H3* and *SREBF1* and enzymes *ACACA* and *FASN* were quantified following acute exposure (6 h). The expression level of *NR1H3* was unchanged in response to fructose, glucose, or the fatty acid combination compared to the vehicle control (Figure 4a). *SREBF1* expression level was also unchanged in response to fructose and glucose but was reduced in response to the fatty acid combination by 36%; however, this difference was not significant (Figure 4b). Additionally, the expression level of *ACACA* was unchanged in response to fructose, glucose, or the fatty acid combination, compared to the vehicle control (Figure 4c). Similarly, the expression level of *FASN* was unchanged in response to fructose and glucose. However, the fatty acid combination significantly reduced *FASN* by 41% (Figure 4d).

## 4. Discussion

The effects of fructose on TG accumulation in liver cells is a common research question that has been addressed with a wide range of methods. Systemic fructose is maintained between 5 and 400 µM [28,29,30] and can increase to millimolar concentrations post-prandially [31,32]. The concentration of fructose detected in the systemic circulation is the best estimate for the concentration that the liver is exposed to, as sampling of portal vein blood is not easy to perform [33]. Reports of post-prandial plasma fructose concentrations vary between studies with most findings in the range of 1–3 mM [32], while one study reported a concentration of 17 mM [31]. In the current study, this range of concentrations was examined, as well as concentrations up to 80 mM, and an interesting negative correlation between fructose concentration and TG accumulation occurred. Similar in vitro studies investigating the effect of fructose in TG and lipid accumulation have treated HepG2 cells with fructose concentrations between 5 and 20 mM. Comparison of results obtained in the current study to these papers is interesting as comparable methods to determine TG concentrations were used, and similar changes in response to positive controls were reported. Yet the response to fructose varied between the studies.

In a study by Lanaspa et al. (2012), a maximum response was detected at 20 mM fructose with a 2.1-fold increase in TGs compared to the vehicle control value of 60 mg/g of soluble protein [16]. While the same concentration of fructose did not cause such a change in the concentration of TGs in the current study, the control TG concentration was comparable (83 mg/g compared to 60 mg/g). Yu et al. (2018) studied a similar range of fructose concentrations (1, 5, and 20 mM) and observed a time- and concentration-dependent increase in TGs using a commercial enzyme-based assay across 12, 24, 48, and 72 h [15]. The TG concentration increased 2-fold at 72 h, similar to results from Lanaspa et al. (2012) [16], compared to the vehicle control value of approximately 70 mg/g of soluble protein. Lanaspa et al. (2012) [16] and Yu et al. (2018) [15] standardised the TG concentration to the total protein concentration in the cell lysates, an approach taken in the current study, and this accounts for changes in cell number in response to sugars. Yu et al. (2018) [15] also reported that the positive control used, palmitic acid (200 µM), increased the TG concentration 2-fold, similar to fructose at 72 h. This suggests fructose is not only a lipogenic substrate but is as lipogenic as fatty acids that are directly incorporated into TGs. This finding was also qualitatively verified with an Oil red O stain, validating increased intracellular lipids following the fructose treatments [15].

Oil red O was also used by Montesano et al. (2020), where 5 mM fructose increased the staining intensity of Oil red O 5-fold compared to the control [17]. Oil red O protocols vary widely and are difficult to compare to TG assays, yet the trend reported adds evidence to the fact that fructose potently increases TGs in HepG2 cells. However, the concentration of glucose in the media of the published studies discussed was not reported. This is a critical factor, as it would indicate if it were the total carbohydrate or fructose that accounted for the increased TG accumulation. Glucose is also a substrate for DNL, and it has been reported as necessary for fructose to induce TG accumulation in HepG2 cells [18], highlighting the importance of not only considering but also reporting the media glucose concentration. Further complicating this, HepG2 cells are often hyperglycolytic, relying on metabolism of glucose rather than oxidative phosphorylation to generate ATP. Therefore, in the current study, HepG2 cells passaged in low- and high-glucose media were tested to determine if excess media glucose affected fructose-induced DNL.

Multiple studies do, however, report the glucose concentration in the culture media. Amorim et al. (2021) investigated lipid accumulation using the fluorescence of Nile red (normalised to cell protein) [19]. HepG2 cells cultured in media with 5 mM glucose were exposed to 10 mM fructose for 1, 6, or 24 h. No effect was seen at 24 h in contrast to the studies mentioned previously. However, this result agrees with experiments conducted in the current study where the fluorescent intensity of Nile red did not change at 48 h in response to 20 mM fructose. Comparable to the current study, Amorim et al. (2021) [19] reported no additional effect of fructose on lipid accumulation in the presence of 0.5 mM palmitic acid and in the presence of a 0.25 mM combination of fatty acids. A 24 h exposure may be too short to observe fructose-induced DNL, and how long it takes for this effect to be seen is not consistent across studies. Additionally, Amorim et al. (2021) [19] confirmed an upregulation of fructokinase following fructose exposure, confirming a result reported by Lanaspa et al. (2012) [16] that fructose metabolism to fructose-1-phosphate occurs in HepG2 cells and results in positive feedback.

In agreement with Amorim et al. (2021) [19], Huggett et al. (2023) reported that in HepG2 cells exposed to a more physiological concentration of fructose (2 and 8 mM), no change in Nile red fluorescent intensity was detected at 48 h [20]. This agrees with results in the current study in which Nile red was used; however, Huggett et al. (2023) [20] used a lower concentration of fructose. Interestingly, the null result of fructose on lipid accumulation was reported in both high (11 mM)- and physiological (5 mM)-glucose media and in the presence, and absence, of a fatty acid mixture (200 µM) [20]. Another key factor is that Huggett et al. (2023) [20] treated HepG2 cells in serum-free media and then added physiological levels of insulin to the media. This was not performed in other studies, or the current experiments, and highlights that fact that variable levels of hormones present in cell culture media can regulate lipogenesis including insulin, glucagon, oestrogens, and thyroid hormones [34]. For example, increased levels of circulating insulin can promote lipogenesis through *SREBP-1c* activation [35], whereas oestrogen reduces lipogenesis by downregulating peroxisome proliferator-activated receptor gamma [36].

Protocols using an acute exposure of up to 72 h are common in in vitro cell culture to investigate mechanisms related to the effect a compound has on cells. However, a disease such as steatotic liver disease that develops over years to decades [37] is poorly modelled with acute exposures. Additionally, in vivo models of steatosis in rodents can introduce fructose in the drinking water for a period of weeks to months [8,9]. The chronic model used in this study followed in vitro protocols that are similar to those used to develop drug-resistant cell lines [38]. This meant cells could be exposed to fructose for a period of 28 days, far exceeding the 72 h limit in most protocols. HepG2 cells remain phenotypically stable for ~15-18 passages; hence, 28 days was the maximum exposure time [39]. This method could be used in future investigations into compounds that have moderate effects on TGs in HepG2 cells. In the current study, fructose and glucose exposure did not lead to an increase in TGs in HepG2 cells over 28 days; instead, they were slightly downregulated at some time points. When taken together with the result that non-physiological concentrations of fructose (80 mM) also reduced TG concentrations, this implies that both high concentrations and chronic exposures to fructose reduce TG concentrations in HepG2 cells. This highlights a limitation of studies reporting a positive effect at 24 h as this may only reflect a rapid change in the cell, whereas chronic exposure may better reflect subtle adaptive change over time, therefore better modelling dietary exposure. Further investigation is needed using primary human hepatocytes that are non-replicating, as carcinoma-derived HepG2 cells divide rapidly and may shunt excess sugars into nucleotide and amino acid synthesis pathways to facilitate cell division, rather than lipid storage. However, in the current study the results were normalised to total cell protein to account for this. Further investigation into fructose-induced modulation of aerobic and glycolytic processes is needed to fully understand how fructose could reduce TG concentrations under certain conditions.

Once it was confirmed that no change in TG concentration in HepG2 cells occurred following a 48 h and 28-day exposure to fructose, the expression levels of transcription factors and enzymes in relation to lipogenesis were investigated. As TG synthesis and mobilisation are tightly regulated [26], a short exposure of 6 h was chosen to examine transient changes that may return to baseline over time. This time point was also chosen to be less than 24 h, where no phenotypic effect was seen in TG accumulation. qPCR assays are the gold standard for detecting changes in the expression level of genes of interest [40]. qPCR was selected over Western blotting to investigate the expression levels of transcription factors and enzymes of interest related to DNL. As the levels of transcription factors and enzymes were examined at 6 h, a sensitive method of detection was required. Hence, qPCR, which detects changes in mRNA upstream of translation to proteins, was selected [41]. The novel primers used were validated to be specific to one target and efficient across a range of cDNA concentrations. Rather than assessing the level of every gene in the pathway of DNL, the central transcription factors LXR-α and SPREBP-1c were selected. ACACA was also selected as it acts upstream of where oleic and palmitic acid are incorporated into TGs, and FAS was selected as it is the rate-limiting enzyme in DNL [42].

Yu et al. (2018) reported in HepG2 cells exposed to 20 mM fructose for 72 h that the expression level of transcription factors *SREBP-1c* and *ChREBP* was upregulated 5-fold and 2-fold, respectively [15]. In the current study, exposure to the same concentration of fructose or 20 mM glucose for 6 h had no effect on the expression of *SREBP-1c*. There was also no effect on *LXR-α*, which regulates the expression of both *SREBP-1c* and *ChREBP*. Yu et al. (2018) [15] also reported that the protein levels of ACACA, FASN, and SCD-1 were all upregulated 2-fold in response to 20 mM fructose. This is in line with the 2.5-fold increase in ATP-citrate lyase, an enzyme upstream of ACACA, in response to 20 mM fructose reported by Lanaspa et al. (2012), whereas in the current study, no change in *ACACA* and *FASN* was seen in response to 20 mM fructose [16]. However, in another investigation, Hirahatake et al. (2011) reported that no change in the mRNA expression or protein level of FAS or ACACA occurred following exposure to 5 mM fructose or glucose for 24, 48, or 72 h [43]. Similarly to TG accumulation, results for gene expression are discordant in the literature. The current experiments agree with Hirahatake et al. (2011) [43]; however, a shorter exposure period was used. The null effect in the current study on the transcription of genes encoding two key transcription factors and two key enzymes involved in DNL confirms and agrees with results previously discussed where no changes in TG accumulation occurred and that fructose has no effect on DNL at a transcriptional or phenotypic level.

The observed reduction in *SREBP-1c* and *FASN* in response to fatty acids confirmed that while no effect was seen in response to fructose, the mechanisms of DNL can be modulated in HepG2 cells. A moderate decrease in the expression level of *FASN* and *SREBP-1c* in response to the combined fatty acid exposure was detected, and agrees with a study by Zhang et al. (2023) in the mouse liver cell line NCTC 1469 [44]. In this study, cells were exposed to a combined fatty acid mixture (750 µM) for 24 h, a 4.5-fold higher concentration than that used in the current study. The fatty acid mixture reduced the expression level of *SREBP-1c* by 20% and *FASN* by 40% compared to 36% and 41%, respectively, in the current study. Zhang et al. (2023) [44] also confirmed that at a protein level, ACACA was reduced as well as ATP-citrate lyase. It was concluded that negative feedback from excessive TG/lipid accumulation downregulated DNL in cells. As oleic acid and palmitic acid are products of DNL, negative feedback to reduce the expression level of transcription factors and enzymes involved in DNL likely also accounts for the results in the current study. Further investigation of protein levels or activity would strengthen this association as any direct interaction with proteins would be uncovered; however, as fructose did not increase the expression of the genes tested, this was not undertaken.

This study used HepG2 cells which have 80% sequence homology with liver tissue and are used in over half of in vitro liver studies [13]. However, low expression of enzymes involved in the pathway of fructose metabolism including aldolase B and predominantly glycolytic metabolism are limitations of using this cell line as a model to investigate effects of fructose in the liver [14]. Specifically, cancer cell lines can utilise glycolysis and shunt sugars into the pentose phosphate pathway to promote cell division, rather than store energy intracellularly, for example, as glycogen and lipid droplets [45]. If this metabolic reprogramming occurs in HepG2 cells, then the findings of the current study support the conclusion that despite being widely used, HepG2 cells are not an adequate model to test fructose in the context of steatosis, rather than the conclusion that fructose is not a lipogenic substrate.

Future investigations into this topic should consider the use of other hepatocyte models including HepaRG cells or primary human hepatocytes which retain 88 and 92% sequence homology, respectively, with liver tissue [13]. These cells have their own set of limitations including cost, limited access, and the technical expertise required to maintain differentiated cells lines. Similarly, 2D monolayer cultures poorly model the unique structures and cell arrangements of the liver. Co-cultures with non-parenchymal cells and 3D cultures such as spheroids, organoids, or organs-on-a-chip present more advanced models where the structure and therefore function of hepatocytes are better preserved. These models may more accurately determine the effect of fructose in TG accumulation and DNL.

## 5. Conclusions

Overall, this study added to evidence that fructose does not affect TG or lipid accumulation in HepG2 cells at acute (48 h) or chronic (28 day) time points. This was true in both low- and high-glucose media and in the presence or absence of fatty acids. qPCR confirmed this null result as key genes related to DNL were not modulated when cells were exposed to fructose.

## Figures and Tables

**Table 1:** Primers used for qPCR.

Gene	Accession Number	Forward Primer (5′–3′)	Reverse Primer (5′–3′)	Efficiency (%)
*NR1H3*	NM_005693.4	CAGGACCAGCTCCAGGTAGA	ATGGGGATGGTGGATGGAGA	99
*SREBF1*	NM_001321096.3	GGGACCACTGTCACTTCCAG	CTTCAAAGCTTCGACGCAGG	96
*ACACA*	NM_198834.3	AGCGAGCAGAAGTCATACGG	AAGGCAGCTCTAGCCCTTTT	99
*FASN*	NM_004104.5	CCATGGCAACGTGATGCTAC	ACGTGGACGGATACTTTCCC	90
*ACTB*	NM_001101.5	TGGCCGAGGACTTTGATTGC	AGGGACTTCCTGTAACAACGCA	94

Primers were designed for the human variant of each gene.

## Data Availability

The raw data supporting the conclusions of this article will be made available by the authors on request.

## Supplementary Materials

The following supporting information can be downloaded at: https://www.mdpi.com/article/10.3390/nu17172762/s1, Figure S1. Effect of oleic acid and palmitic acid on cell number in HepG2 cells. Figure S2. Effect of fructose on protein concentrations. Figure S3. Effect of sugars alone, or in combination with a fatty acid mixture on protein concentrations.

## Abbreviations

The following abbreviations are used in this manuscript:

MASLD	Metabolic (dysfunction)-associated steatotic liver disease
TG	Triglyceride
DNL	De novo lipogenesis
DMEM	Dulbecco’s modified Eagle medium
SRB	Sulforhodamine B
BSA	Bovine serum albumin
