# Supplementary material for: Effects of Fructose on Features of Steatotic Liver Disease in HepG2 Cells
Source: Nutrients. 2025 Aug 26;17(17):2762. doi: 10.3390/nu17172762 (PMC12430389; doi:10.3390/nu17172762)
Supplement: Supplementary file 1 [file nutrients-17-02762-s001.zip › nutrients-3790711-supplementary.pdf]

Supplementary File

Effects of fructose on features of steatotic liver disease in HepG2 cells.

Matthew T Howes [1,*], Jessie King [1] and Rhonda J Rosengren [1]

[1] Department of Pharmacology and Toxicology, University of Otago, 9016, Dunedin, New Zealand; mhowes63@gmail.com (M.H); jessie.king@otago.ac.nz (J.K.); rhonda.rosengren@otago.ac.nz (R.R.)
* Correspondence: mhowes63@gmail.com

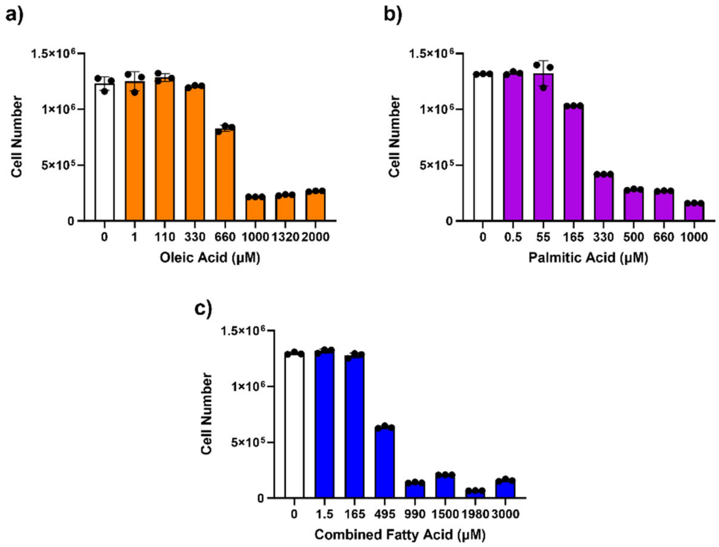

Figure S1. Effect of oleic acid and palmitic acid on cell number in HepG2 cells. Cells were plated at 3.5 x 10^5 cells per well in 6 well plates and exposed to varying concentrations of a) BSA-conjugated oleic acid, b) BSA-conjugated palmitic acid, c) or a combination of both for 72 h. Cell number was determined using an SRB assay with absorbance read at 510 nm and linear regression was used to determine cell number relative to a standard curve. Results are expressed as the mean from n = 1 replicate performed in triplicate.

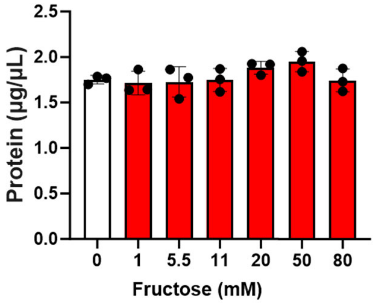

Figure S2. Effect of fructose on protein concentrations. HepG2 cells plated in 6-well plates at 3.5 x 10^6 cells/well were exposed to a range of fructose concentrations for 72 h. Cell lysates were incubated with the Pierce BCA protein reagent and absorbance at 562 nm was used to determine the protein concentration relative to a standard curve using linear regression. Columns represent the mean ± SD from three independent experiments performed in duplicate. Statistical significance was tested using a one-way ANOVA. No difference between groups was detected.

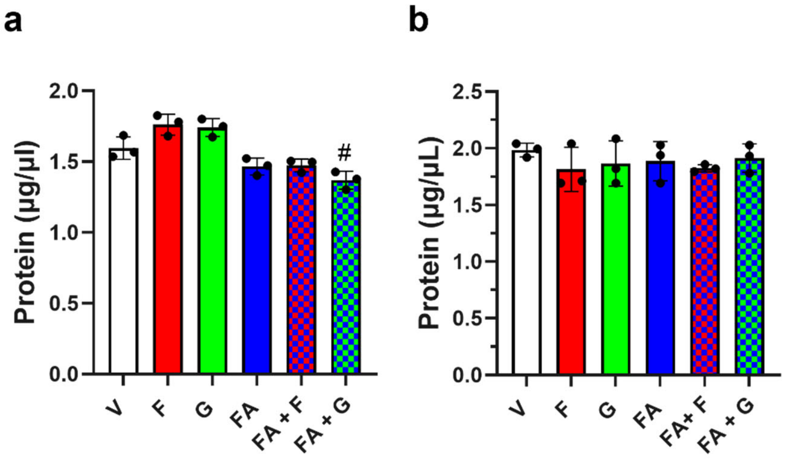

Figure S3. Effect of sugars alone, or in combination with a fatty acid mixture on protein concentrations. HepG2 cells cultured in a) low glucose media (5.5 mM) or b) high glucose media (25.5 mM) were treated with vehicle (V), fructose (F, 20 mM), or glucose (G, 20 mM) alone or in combination with oleic and palmitic acid (FA, 110 µM / 55 µM) for 48 h. The protein concentration in cell lysates were determined using the Pierce BCA protein reagent. Columns represent the mean ± SD from three independent replicates performed in duplicate. Statistical significance was analysed using a one-way ANOVA with differences between groups determined using a Bonferroni multiple comparison *post-hoc* test. # significantly decreased compared to F and G, $p < 0.05$.
